# A New Alignment-Free Whole Metagenome Comparison Tool and Its Application on Gut Microbiomes of Wild Giant Pandas

**DOI:** 10.3389/fmicb.2020.01061

**Published:** 2020-06-16

**Authors:** Jiuhong Dong, Shuai Liu, Yaran Zhang, Yi Dai, Qi Wu

**Affiliations:** ^1^State Key Laboratory of Mycology, Institute of Microbiology, Chinese Academy of Sciences, Beijing, China; ^2^Institute of Physical Science and Information Technology, Anhui University, Hefei, China; ^3^Key Laboratory of Animal Ecology and Conservation Biology, Institute of Zoology, Chinese Academy of Sciences, Beijing, China; ^4^University of Chinese Academy of Sciences, Beijing, China

**Keywords:** whole metagenome comparison, k-mer frequencies, reverse complementary sequence, cosine distance, wild giant pandas, gut microbiomes

## Abstract

The comparison of metagenomes is crucial for studying the relationship between microbial communities and environmental factors. One recently published alignment-free whole metagenome comparison method based on k-mer frequencies, Libra, showed higher resolutions than the present fastest method, Mash, on whole metagenomic sequencing reads, but it did not perform as well on the assembled contigs. Here, we developed a new alignment-free tool, KmerFreqCalc, for the comparison of the whole metagenomic data, which first calculated the frequencies of both forward and reverse complementary sequences of k-mers like Mash and then computed the cosine distance between the samples based on k-mer frequency vectors like Libra. We applied KmerFreqCalc on the assembled contigs of the gut microbiomes of wild giant pandas and compared the results to Libra and Mash. The results indicated that KmerFreqCalc was able to detect the subtle difference between giant panda samples caused by seasonal diet change, showing better clustering than Libra and Mash. Therefore, KmerFreqCalc has high resolution and accuracy for assembled contigs, being very suitable for comparison of samples with low dissimilarity.

## Introduction

The comparison of metagenomes is crucial for studying the relationship between microbial communities and environmental factors. Traditionally, the dissimilarity between gut microbiomes is assessed based on the microbial diversity or the abundance of genes under special functional categories. The former relies on the 16S rRNA gene or the whole metagenomic data, classifying the bacterial groups and the archaeal ones at the genus level to the species level or the subspecies level, for example, the work on mammals (Ley et al., 2008), vertebrates (Youngblut et al., 2019), and the giant pandas (Zhu et al., 2011; Xue et al., 2015; Wu et al., 2017). The latter uses the whole metagenomic data, first mapping the short reads to the known genes or the pathways in the existing databases, such as NR, KEGG, or IMG, and then comparing their abundances between the samples based on the mapped functional categories, for example, the works on mammals (Muegge et al., 2011), whales (Sanders et al., 2015), and giant pandas (Guo et al., 2018; Zhu et al., 2018). Both methods are based on the alignment of sequences to the reference databases, thus using only a fraction of the whole metagenomes due to the limitation of the availability and the completeness of the existing databases.

In order to fully use the genomic information, we can alternatively take a class of alignment-free approaches based on the frequency of k-mers (or k-tuples, k-grams) which are thought to represent the sequence signature of the genomes. Over the past decade, such methods have proved to be valid for comparing genomic sequences of the individual organisms (Qi et al., 2004) and microbiomes (Jiang et al., 2012). Now, several algorithms have been developed for the comparison of the whole metagenomes based on k-mer frequencies, such as COMMET (Maillet et al., 2014), Simka (Benoit et al., 2016), Mash (Ondov et al., 2016), and Libra (Choi et al., 2019). But due to the different distance calculation strategies, each of them has its own advantages and disadvantages. For example, Mash, the fastest method considered, reduces large sequences and sequence sets to small, representative sketches, from which global mutation distances can be rapidly estimated using Jaccard similarity. But the subset of unique k-mers might lead to an unrepresentative k-mer profile of the samples, so Mash showed lower resolution on the whole metagenome shotgun sequencing (WMGS) reads than another algorithm, Libra, which calculates cosine similarity by default based on k-mer frequencies, using both sequence composition and abundance of sequencing reads (Choi et al., 2019). But when analyzing the assembled contigs, Libra showed a lower resolution than Mash (Choi et al., 2019). For such a result, Choi et al. (2019) have addressed that libra required reads rather than contigs to perform accurately. But after carefully comparing the analysis process of these two methods, we found that considering the reverse complementary sequences of k-mers might be the key factor for the higher resolution of Mash on the assembled contigs.

Here, by absorbing the advantage of Mash and Libra, we developed a new alignment-free algorithm, KmerFreqCalc, for the comparison of the whole metagenomes. This algorithm calculates the frequencies of all possible k-mers at the specific length and their reverse complementary sequences like Mash, based on which the cosine distance between the paired samples is computed like Libra. Hence this new method is expected to have a better performance than Mash and Libra on the assembled contigs. To prove it, we applied KmerFreqCalc on two recently published datasets of gut microbiomes from wild giant pandas (*Ailuropoda melanoleuca*; Wu et al., 2017; Zhu et al., 2018), and compared the results to Mash and Libra. We chose the two datasets as the testing data for two reasons. One was that both datasets have performed WMGS, but neither did the whole metagenome comparison. The other one is that they sampled from different habitats, Qinling (Wu et al., 2017) and Xiangxiangling (Zhu et al., 2018) mountains, but until now no comparing analysis has been performed between the gut microbiomes of giant pandas living in the two isolated habitats. Therefore, the application of the alignment-free tools on these two datasets not only can test the improvement of KmerFreqCalc, but also will provide a new perspective of the gut microbiomes of wild giant pandas.

## Materials and Methods

### KmerFreqCalc Detailed Description

**FIGURE 1:**
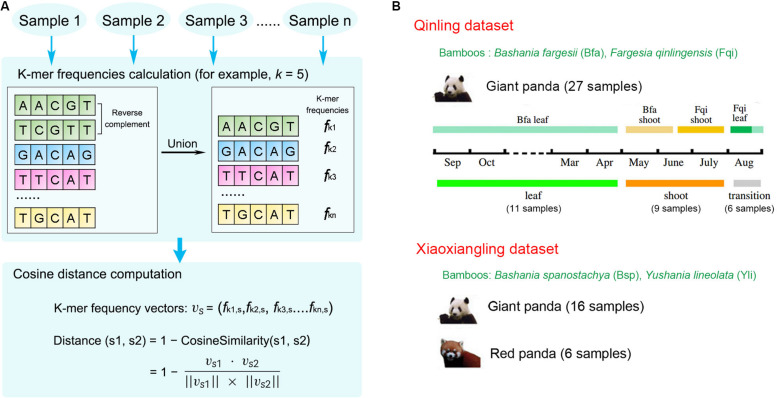
KmerFreqCalc workflow and the data description. **(A)** The overview of the KmerFreqCalc workflow: (1) calculating the k-mer frequencies of each samples, (2) computing the distance between paired samples. **(B)** Two published metagenomic datasets including samples from wild giant pandas. The figure about partition of three stages and four food categories during 1 year in Qinling Mountains was adapted from “Seasonal variation in nutrient utilization shapes gut microbiome structure and function in wild giant pandas” (Wu et al., 2017) with permission. Above the time line is the four food categories. Below the time line is the three stages, in which the leaf, shoot, and transition stages are shown in green, orange, and gray, respectively.

We developed KmerFreqCalc^1^, an implementation of the algorithm that calculates k-mer frequencies of the metagenomic assembled contigs, from which the cosine distance between the paired samples is estimated (Figure 1A). In detail, given a specific k-mer length (also called k-mer size, abbreviated as “*k*” hereafter), the frequencies of forward and reverse complementary sequences of all possible k-mers are used to maximumly characterize the assembled contigs, and then the cosine distance between the samples were calculated based on the actual k-mer frequency vectors as previously described (Qi et al., 2004; Choi et al., 2019).

### Data Description

Two recently published datasets have been analyzed in this study (Figure 1B and Supplementary Table S1).

#### Qinling Dataset (QIN Dataset)

The WMGS reads of the giant pandas living in the Qinling Mountains were downloaded from the GSA database^2^ under bioproject accession no. PRJCA000366, including 27 samples from 6 individuals (Wu et al., 2017). The fecal samples were collected by tracking the GPS-collared giant pandas in 2012 and 2013. Based on feeding behavior and diet, three forage stages were identified: the leaf stage (11 samples), shoot stage (10 samples), and the transition stage (6 samples). From January to May, giant pandas feed on *Bashania fargesii* (abbreviated as Bfa hereafter) leaves at low elevations. From May to July, they switch to Bfa shoots. In the middle of July, when Bfa shoots grow too crude, giant pandas feed on *Fargesia qinlingensis* (abbreviated as Fqi hereafter) shoots at higher elevations. For a short period in August, they eat Fqi leaves, then descend to feed on Bfa leaves again until December.

Illumina Genome Analyzer was used for metagenomic shotgun sequencing (Wu et al., 2017). The WMGS reads were quality filtered with Trimmomatic (Bolger et al., 2014) with parameters ILLUMINACLIP: TruSeq2-PE.fa:2:30:12:1:true LEADING:3 TRAILING:3 MAXINFO:40:0.996 MINLEN:36, and then filtered with host genome data to facilitate the removal of the host sequence. The generated clean reads were assembled to generate long contig sequences with MegaHIT (Li D. et al., 2015).

#### Xiaoxiangling Dataset (XXL Dataset)

The WMGS assembled contigs of bamboo-eating pandas in Xiaoxiangling Mountains were downloaded from figshare at https://doi.org/10.6084/m9.figshare.6303713, including 22 samples (Zhu et al., 2018). Sixteen fresh fecal samples from giant pandas and 6 from red pandas (*Ailurus fulgens*) were collected from 2012 to 2016. The dominant compositions of the fresh feces (leaves, stems, or shoots from *Bashania spanostachya* and *Yushania lineolata*) were recorded. Nine out of the 16 giant panda samples were from 4 GPS-collared individuals translocated to this mountain [Luxin (LX), Zhangxiang (ZX), Taotao (TT), and Huajiao (HJ)].

Illumina HiSeq 2500 platform was used for metagenomic shotgun sequencing (Zhu et al., 2018). The WMGS reads were quality filtered using custom Perl scripts and Trimmomatic (Bolger et al., 2014). Then raw short reads were compared against the host genome to facilitate the removal of host genomic sequences. The resultant clean, high-quality reads were assembled to generate contigs using the SOAPdenovo assembler (Li et al., 2010).

### Alignment-Free Whole Metagenome Comparisons

The KmerFreqCalc was compared with Mash and Libra in the whole metagenome comparisons of the two datasets including samples from wild giant pandas. As we know, the k-mer length was an important factor for the accuracy of alignment-free comparisons, in other words, larger values of *k* result in fewer matches due to sequencing errors and fragmentary metagenomic data, while smaller ones give less information about the sequence similarities (Choi et al., 2019). Hence *k* was a configurable parameter in all three algorithms. We performed our analyses with *k* equal to 15, 17, 19, and 21. Among the values, 21, the default parameter of Mash and Libra, had been reported to be at the inflection point where the k-mer matches move from random to a representative of the read content and is generally resilient to sequencing error and variation (Kurtz et al., 2008; Hurwitz et al., 2014). The other three values were selected as alternatives for optimization. Given a *k* value, Mash and Libra were run with default parameters. Once the calculated distance data were available, neighbour joining (NJ) phylogenetic tree and principal coordinates analysis (PCoA) were used to illustrate the results. Based on cosine distance data with negative normality test results, rank-sum tests were used to determine whether the differences between groups were significant.

## Results

### Whole Metagenome Comparisons of all Samples in the QIN Dataset

**FIGURE 2:**
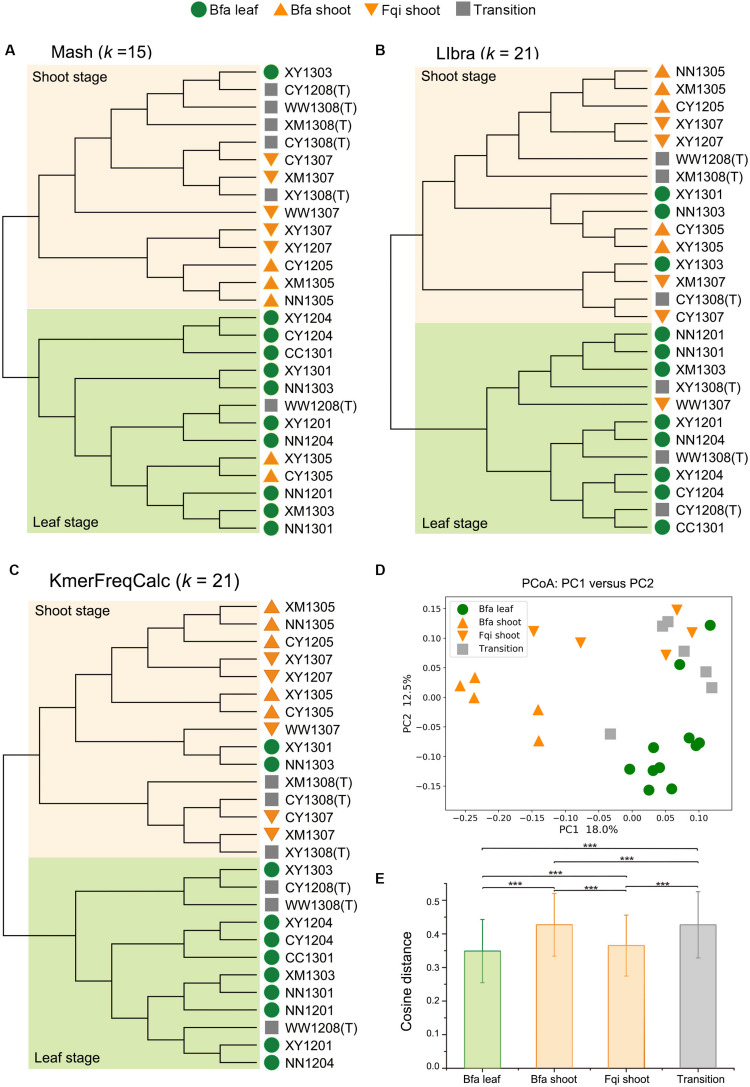
Whole metagenome comparisons of samples in QIN dataset using Mash, Libra and KmerFreqCalc. In NJ tree, two clades of the shoot stage and leaf stage are highlighted with lightorange and lightgreen, respectively. The diet stages are indicated by green circles (Bfa leaf), orange regular triangles (Bfa shoot), orange inverted triangles (Fqi shoot) and gray squares (Transition). **(A)** NJ tree based on the distance calculated by Mash (*k* = 15). **(B)** NJ tree based on the distance calculated by Libra (*k* = 21). **(C)** NJ tree based on the distance calculated by KmerFreqCalc (*k* = 21). **(D)** PCoA analysis using the cosine distance calculated by KmerFreqCalc (*k* = 21). **(E)** Variations in different stages (Bfa leaf, Bfa shoot, Fqi shoot and transition) were determined by cosine distance calculated by KmerFreqCalc (*k* = 21). Mean values ± standard errors of the means are shown. ***p < 0.001 (Rank-sum test).

For the QIN dataset, all three alignment-free algorithms showed ability to cluster most samples by seasonal, as such, samples from the leaf and shoot stages clustered into two groups despite some sporadic data points, while samples from the transition stage occurred in two clusters (Supplementary Figures S1–S3). However, KmerFreqCalc showed a higher resolution than Mash and Libra. Mash performed its best resolution, with *k* equal to 15, but clustered 2 shoot stage samples in the leaf stage clade and 1 leaf in the shoot one (Figure 2A). Libra showed its best resolution with *k* equal to 21, with 1 shoot stage sample in the leaf stage clade and 3 leaf in shoot stage one (Figure 2B). The best resolution of KmerFreqCalc was obtained when *k* was equal to 21, with only 2 leaf stage samples in the shoot stage clade (Figure 2C). Therefore, further PCoA analysis and rank-sum tests were based on the distance calculated by KmerFreqCalc, with *k* equal to 21. Better than the previous analysis based on the 16S rRNA gene (Wu et al., 2017), in PCoA analysis, samples in shoot stage formed two distinct clusters, Bfa shoot and Fqi shoot (Figure 2D). The rank-sum tests indicated that the variations between different stages were significant (*p* < 0.001) (Figure 2E).

### Whole Metagenome Comparisons Between all Samples in the XXL Dataset

**FIGURE 3:**
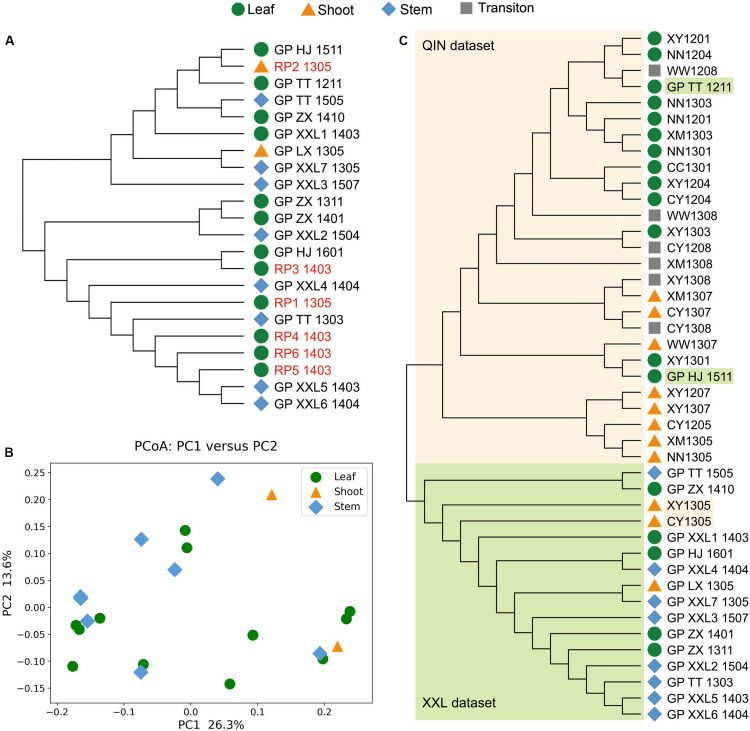
Whole metagenome comparisons of samples in XXL dataset and all samples from giant pandas in two datasets using KmerFreqCalc (*k* = 21). **(A)** NJ tree of samples in XXL dataset. **(B)** PCoA analysis using the cosine distance calculated by KmerFreqCalc (*k* = 21). **(C)** NJ tree of all samples from giant pandas in two datasets, clearly indicating two groups of QIN dataset and XXL dataset highlighted with lightorange and lightgreen, respectively.

For the XXL dataset, all analyses using three alignment-free algorithms clustered samples from giant pandas and red pandas together (Figures 3A,B and Supplementary Figures S4–S6A). The gut microbiomes of giant pandas and red pandas showed no significant difference (*p* > 0.05). The samples with shoots, stems, or leaves of bamboos in feces also showed no significant difference (*p* > 0.05), intermingling together in NJ-tree.

### Combined Comparisons of the Whole Gut Metagenomes From Giant Pandas in Qinling and Xiaoxiangling Mountains

In order to identify the variation of gut microbiomes from the wild giant pandas living in different habitats, all samples of the giant pandas from two datasets were compared together using three alignment-free algorithms (Supplementary Figures S7–S9). The results showed that the samples in QIN and XXL dataset clearly clustered into two groups in various *k* values, despite some sporatic samples (Figure 3C).

## Discussion

K-mer frequencies have been extensively used in alignment-free methods for comparing genomes or metagenomes. Due to the different strategies of distance calculation, each existing method has its own merits. By absorbing the advantages of two good algorithms, Mash and Libra, we developed a new method, KmerFreqCalc. Its characters are: (1) considering the frequencies of both forward and reverse complementary sequence of k-mers like Mash, which can give a representative profile of the metagenome assembled contigs and (2) calculating the cosine distance between paired samples based on the k-mer frequency vectors like Libra, which takes in account all the sequence signatures contrasting to the subset of unique k-mers used by Mash. Based on this, when using the assembled contigs, KmerFreqCalc can obtain higher resolution clustering of samples than Mash and Libra. The application of these three algorithms on the gut microbiomes of the wild giant pandas has confirmed this improvement. Meanwhile, our whole metagenome comparisons brought some new sight of the panda gut microbiomes. The following is the detailed explanation.

Giant pandas are bamboo specialists that evolve from carnivores, possessing a gastrointestinal tract typical of carnivores (Wei F. et al., 2015). Their gut microbiomes closely resemble that of other carnivores (Ley et al., 2008), possessing few special gut bacteria because of their exclusively bamboo diet (Zhu et al., 2011). Today, wild giant pandas live in six relatively isolated habitats in western China mountain area (Qinling, Minshan, Qionglai, Liangshan, Daxiangling, and Xiaoxiangling) (Zhao et al., 2013), where they forage on different bamboo species and different parts of the bamboo plant in different seasons (Wei W. et al., 2015). Wu et al. (2017) have indicated that the gut microbiomes of wild giant pandas living in Qinling Mountains seasonally changed along with the diet change. Zhu et al. (2018) investigated the potential mechanism of detoxification of cyanide compounds by gut microbiomes of bamboo-eating pandas living in Xiangxiangling mountains. The WMGS data from the last two studies mentioned above (Wu et al., 2017; Zhu et al., 2018) was chosen to be testing dataset of our study. The results were very inspiring.

Firstly, because the samples from the Qin dataset have been clustered into distinct groups by seasonal diet based on the 16S rRNA gene data, this dataset is well suited for comparing KmerFreqCalc with Mash and Libra. The results showed that KmerFreqCalc had higher resolutions of the samples in different diet stages than Libra and Mash, clustering the samples from shoot stage and leaf one into two distinction groups. This indicated that reverse complementary sequence of k-mer was important for the comparison of metagenomes on the assembled contigs. In addition, better than previous study on 16S rRNA gene data, the variations between two similar stages, Bfa shoot and Fqi shoot, were revealed by KmerFreqCalc. This suggested that KmerFreqCalc has high resolution and sensitivity, so it can be a good choice for estimating the genetic distance of metagenomes, especially the samples with low dissimilarity in time series.

Second, as we know, diet drives convergence in gut microbiome across mammalian phylogeny (Muegge et al., 2011; Youngblut et al., 2019). Adapting to a specialized bamboo diet not only promotes the genetic convergence between giant panda and red pandas (Hu et al., 2017), but also the gut metagenomic convergence (Li Y. et al., 2015; Zhu et al., 2018). Our analyses on the XXL dataset confirmed the high similarity of the gut microbiomes of bamboo-eating pandas in Xiaoxiangling Mountains, which indicated that besides the high resolution and the sensitivity, KmerFreqCalc had low false positive. Panda samples from the Xiaoxiangling Mountains with different components, including stems, leaves, shoots in feces were not further divided into independent clades. This might be due to the asymmetrical sample size (leaf: 12 samples; stem: 8 samples; and shoot: 2 samples), the weak variation of the diet or the different genetic background of the native pandas and the translocated pandas.

Finally, the combined comparison of the metagenomic data from the giant pandas living in different habitats (Qinling and the Xiaoxiangling Mountains) presented two distinct clusters of the samples from XXL and QIN, except some sporatic data. It is highly likely due to the significant difference between the gut microbiomes of the wild giant pandas living in Qinling and the Xiaoxiangling mountains, considering of the following three reasons. The first one is the population differentiation due to the genetic adaptation to their environments (Zhao et al., 2013). The second one is the different bamboo species the pandas have in two habitats (Figure 1B). And the last one is the different environmental microbiomes in the two habitats. However, it has to be mentioned that the sequencing coverage and the quality of assembled contigs probably have affected the analysis results, so the discrepancy between gut microbiomes of the wild giant pandas living in different habitats should be kept as an open question till more data, especially the raw data with comparable coverage, was available.

## Conclusion

The work has developed a new alignment-free method for comparing the whole metagenomes with high resolution and accuracy and applied this method on the gut microbiome comparison of the wild giant pandas. The results have confirmed the influence of diet and habitat on gut metagenomes of wild giant pandas. However, the new method for comparing the function of metagenomes through the association between k-mers and genes remains to be developed, which we believe will bring some new perspectives to this topic.

## Data Availability Statement

The two metagenome datasets used in this study both were published. Sequencing reads of QIN dataset was deposited in the GSA database (http://bigd.big.ac.cn/) under bioproject accession no. PRJCA000366. Assembled contigs of XXL dataset was deposited in figshare at https://doi.org/10.6084/m9.figshare.6303713.

## Ethics Statement

This study was performed on the published metagenomic data based on the sequencing of fecal DNA of animals, so it did not involve regulated animals and should be exempt from ethics approval.

## Author Contributions

JD and QW designed the research. JD performed the research. SL developed the algorithm. JD and YZ prepared the figures. YD performed the statistical analyses. JD wrote the manuscript. All authors read and approved the final manuscript.

## Conflict of Interest

The authors declare that the research was conducted in the absence of any commercial or financial relationships that could be construed as a potential conflict of interest.

## Footnotes

1 https://github.com/LDS-axel/KmerFreqCalc

2 http://bigd.big.ac.cn/
