# Supplementary material for: A New Alignment-Free Whole Metagenome Comparison Tool and Its Application on Gut Microbiomes of Wild Giant Pandas
Source: Front Microbiol. 2020 Jun 16;11:1061. doi: 10.3389/fmicb.2020.01061 (PMC7309450; doi:10.3389/fmicb.2020.01061)
Supplement: Supplementary file 1 [file Data_Sheet_1.PDF]

Supplementary Material

Table S1 Detailed information of the metagenomic data in QIN and XXL datasets.

Supplementary Figures

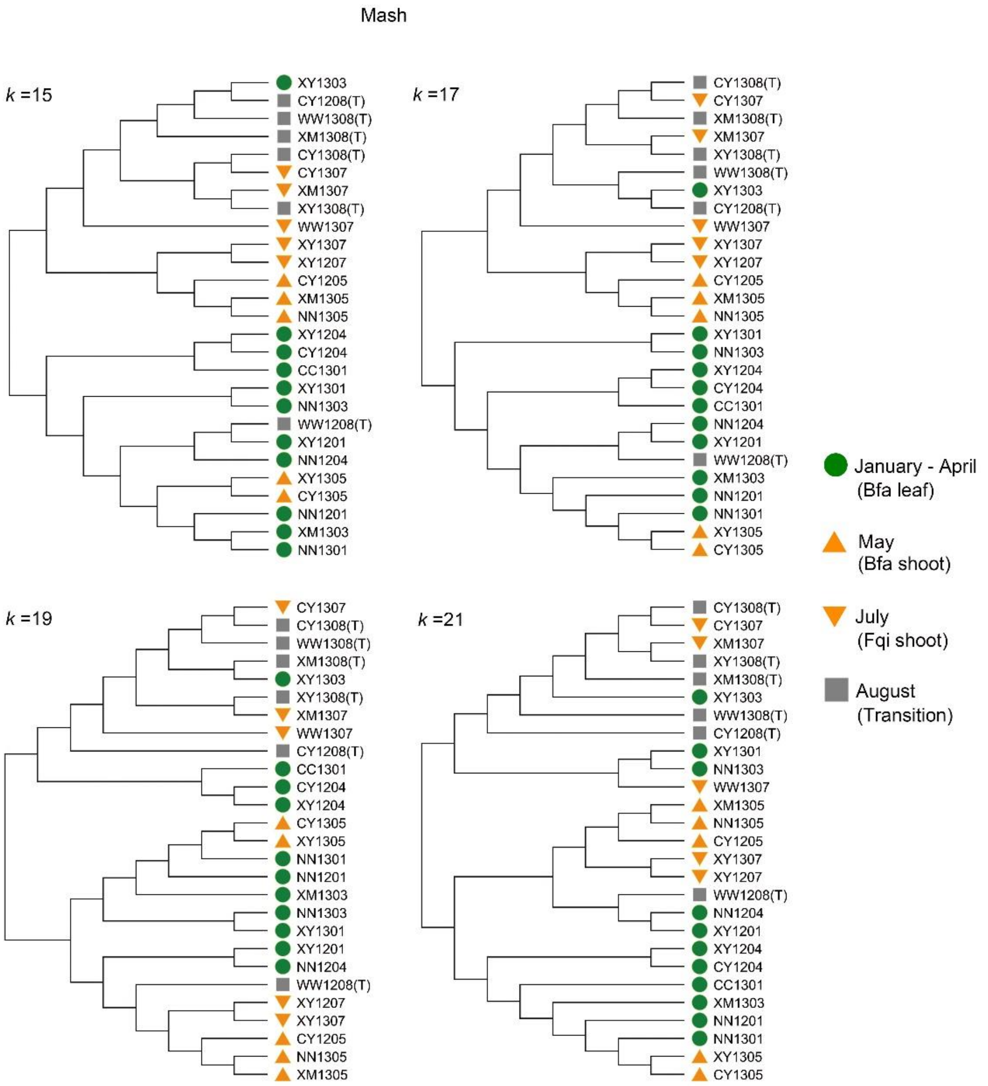

Figure S1 NJ-analysis of samples in QIN dataset based on Jaccard distance calculated by Mash with *k* = 15, 17, 19 and 21.

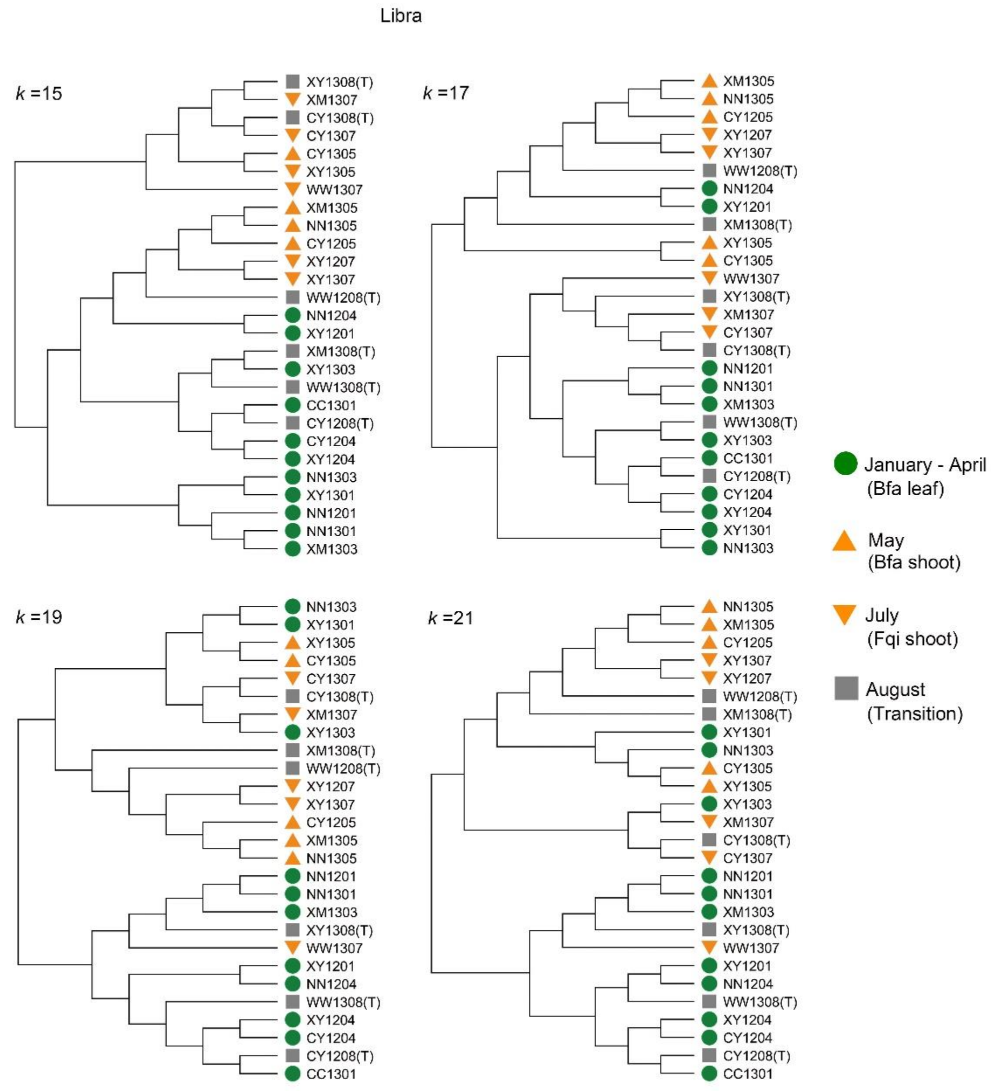

Figure S2 NJ-analysis of samples in QIN dataset based on Jaccard distance calculated by Libra with *k* = 15, 17, 19 and 21.

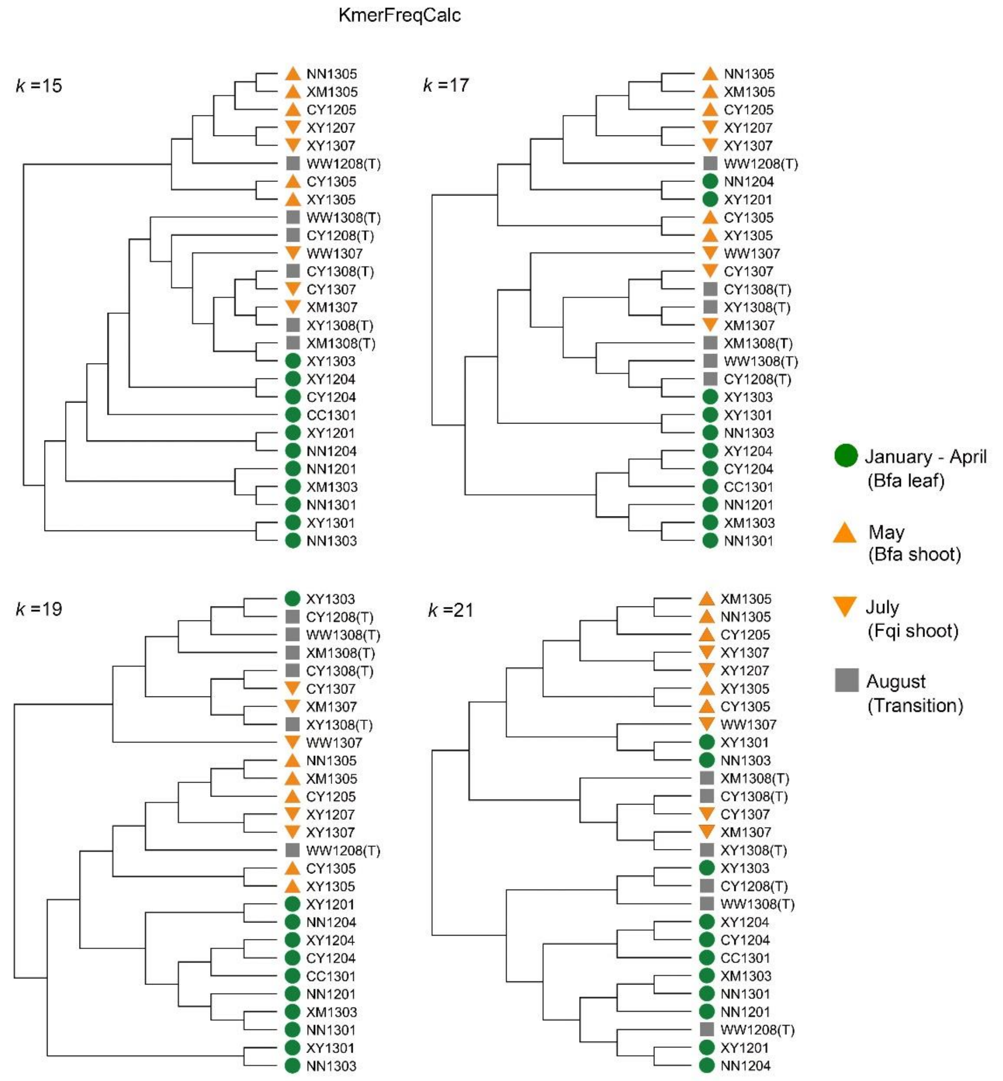

Figure S3 NJ-analysis of samples in QIN dataset based on Jaccard distance calculated by KmerFreqCalc with k = 15, 17, 19 and 21.

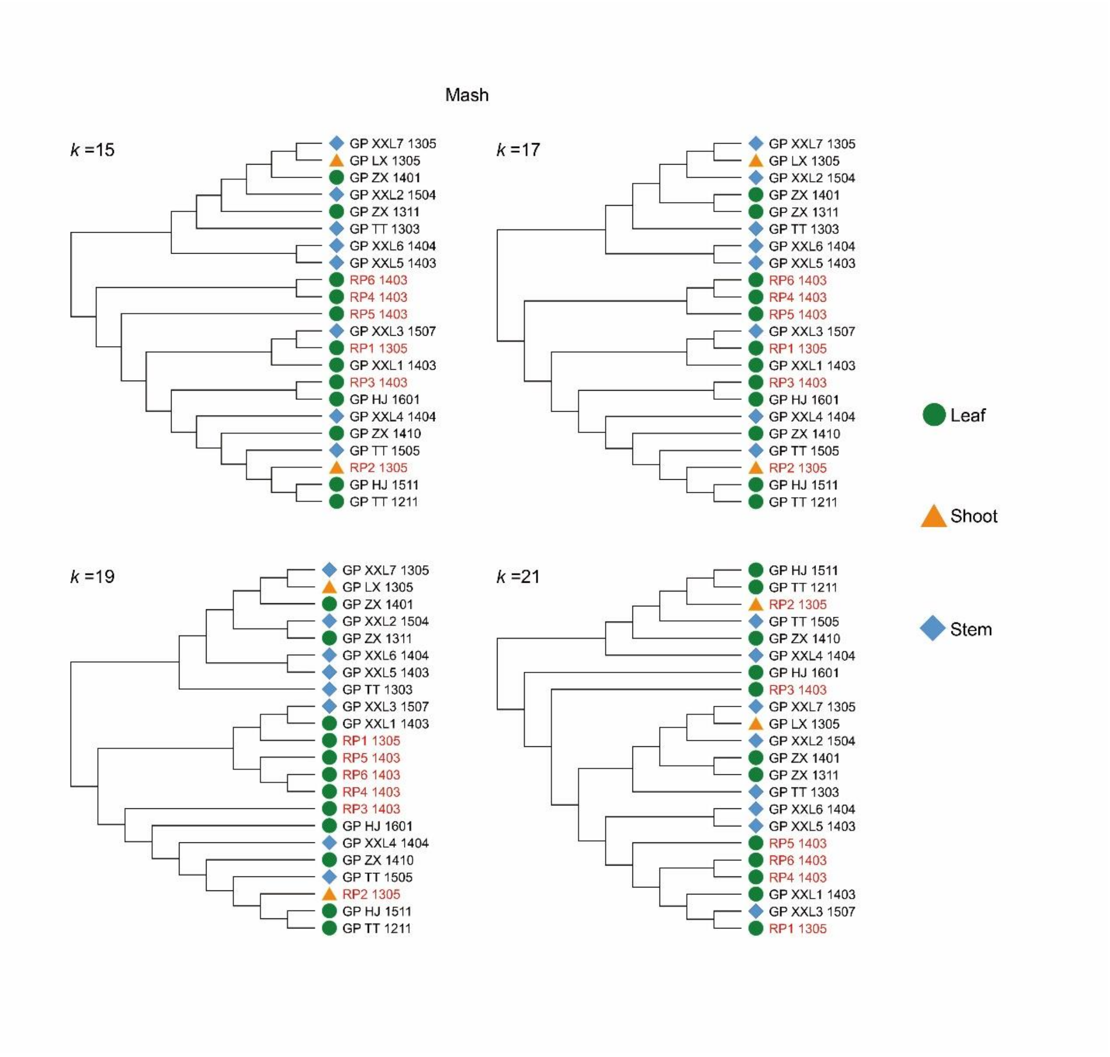

Figure S4 NJ-analysis of samples in QIN dataset based on Jaccard distance calculated by Mash with *k* = 15, 17, 19 and 21.

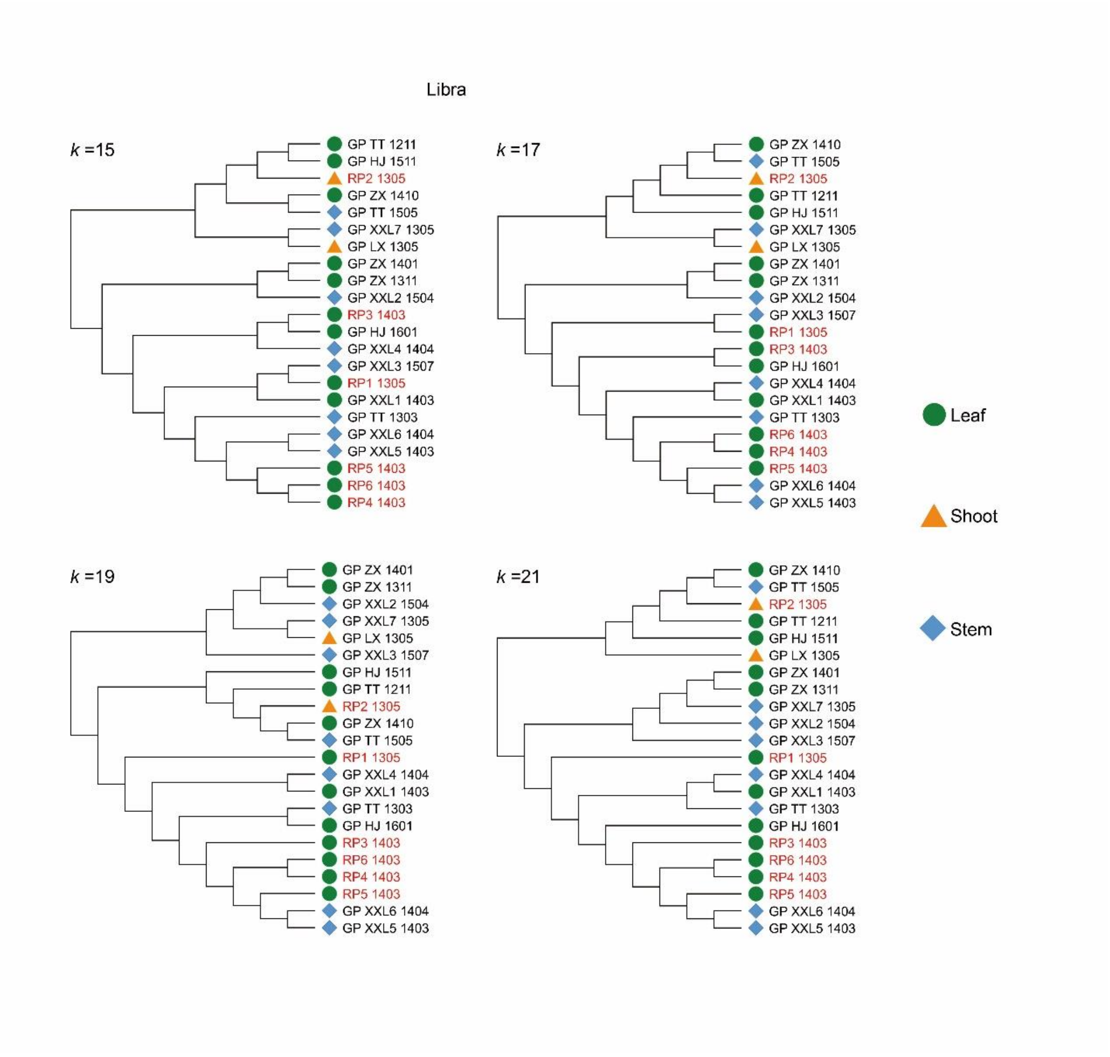

Figure S5 NJ-analysis of samples in QIN dataset based on Jaccard distance calculated by Libra with *k* = 15, 17, 19 and 21.

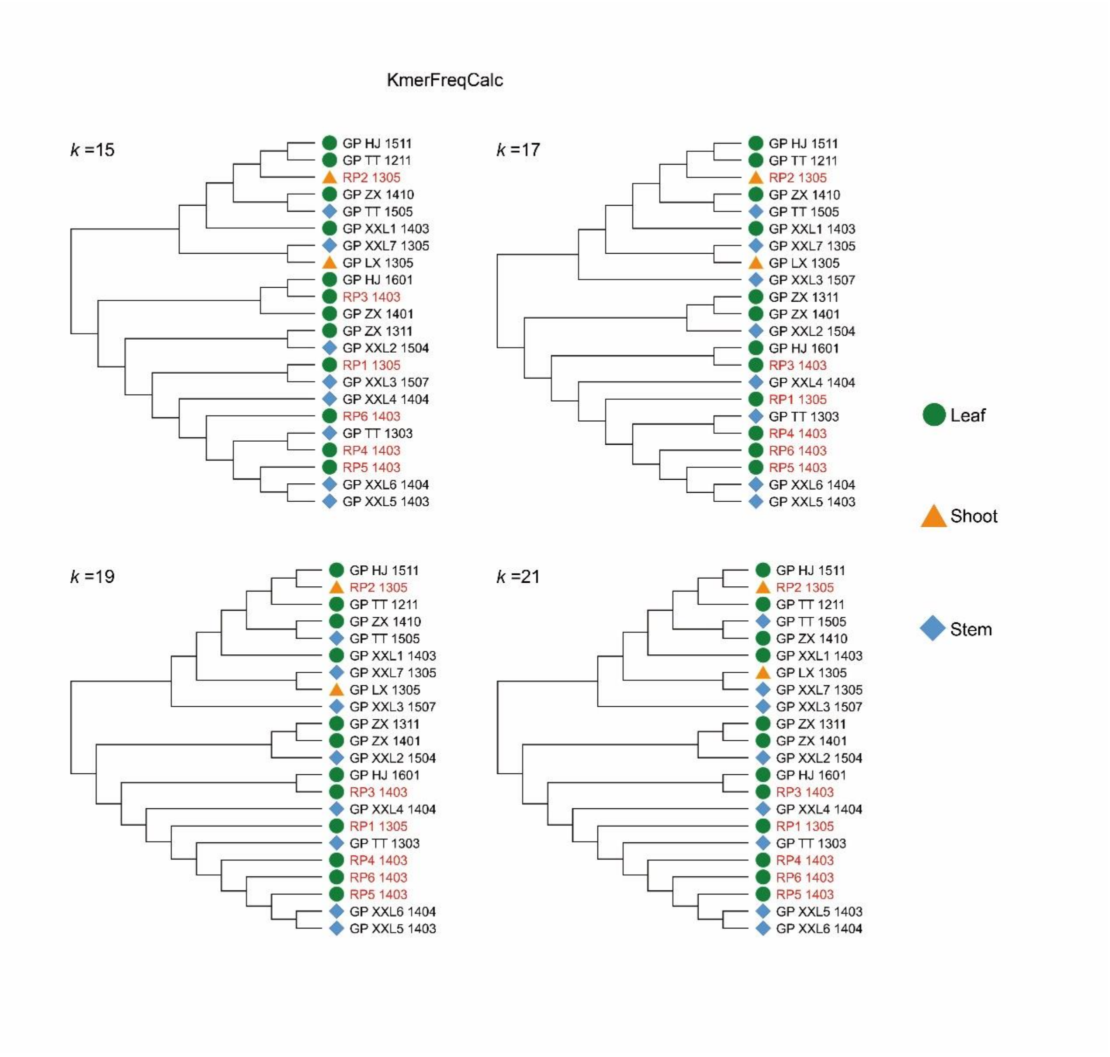

Figure S6 NJ-analysis of samples in QIN dataset based on Jaccard distance calculated by KmerFreqCalc with *k* = 15, 17, 19 and 21.

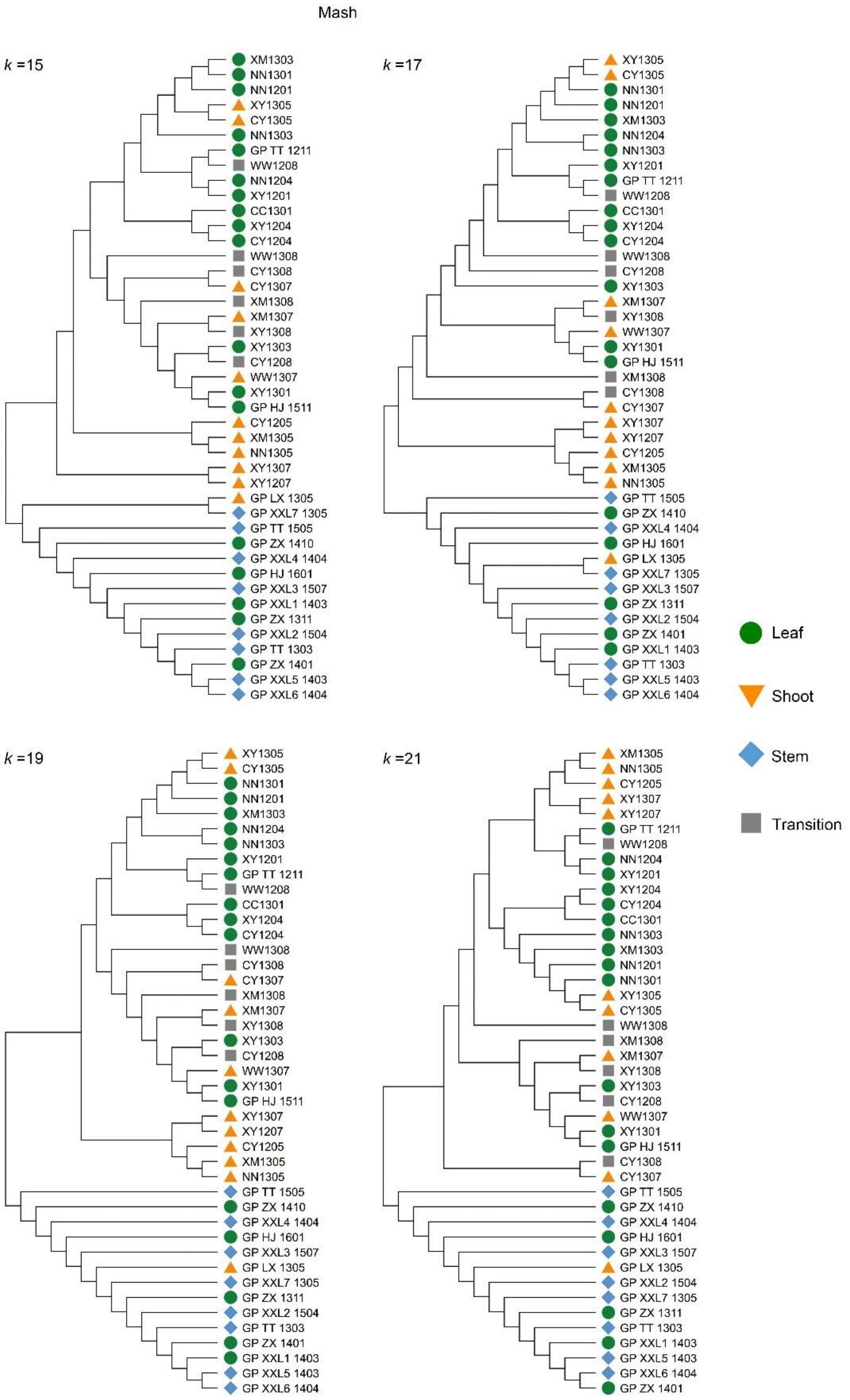

Figure S7 NJ-analysis of samples in QIN dataset based on Jaccard distance calculated by Mash with k = 15, 17, 19 and 21.

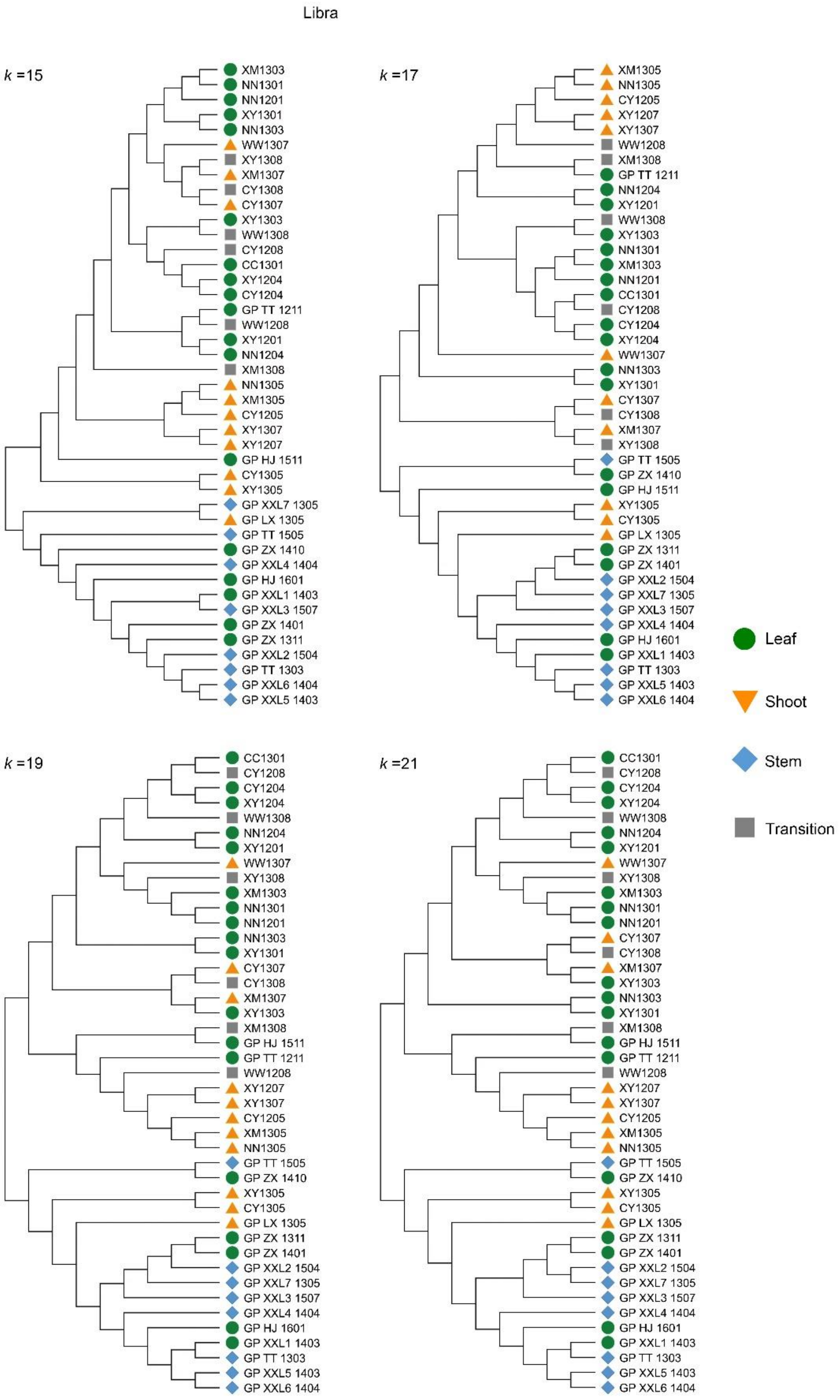

Figure S8 NJ-analysis of samples in QIN dataset based on Jaccard distance calculated by Libra with k = 15, 17, 19 and 21.

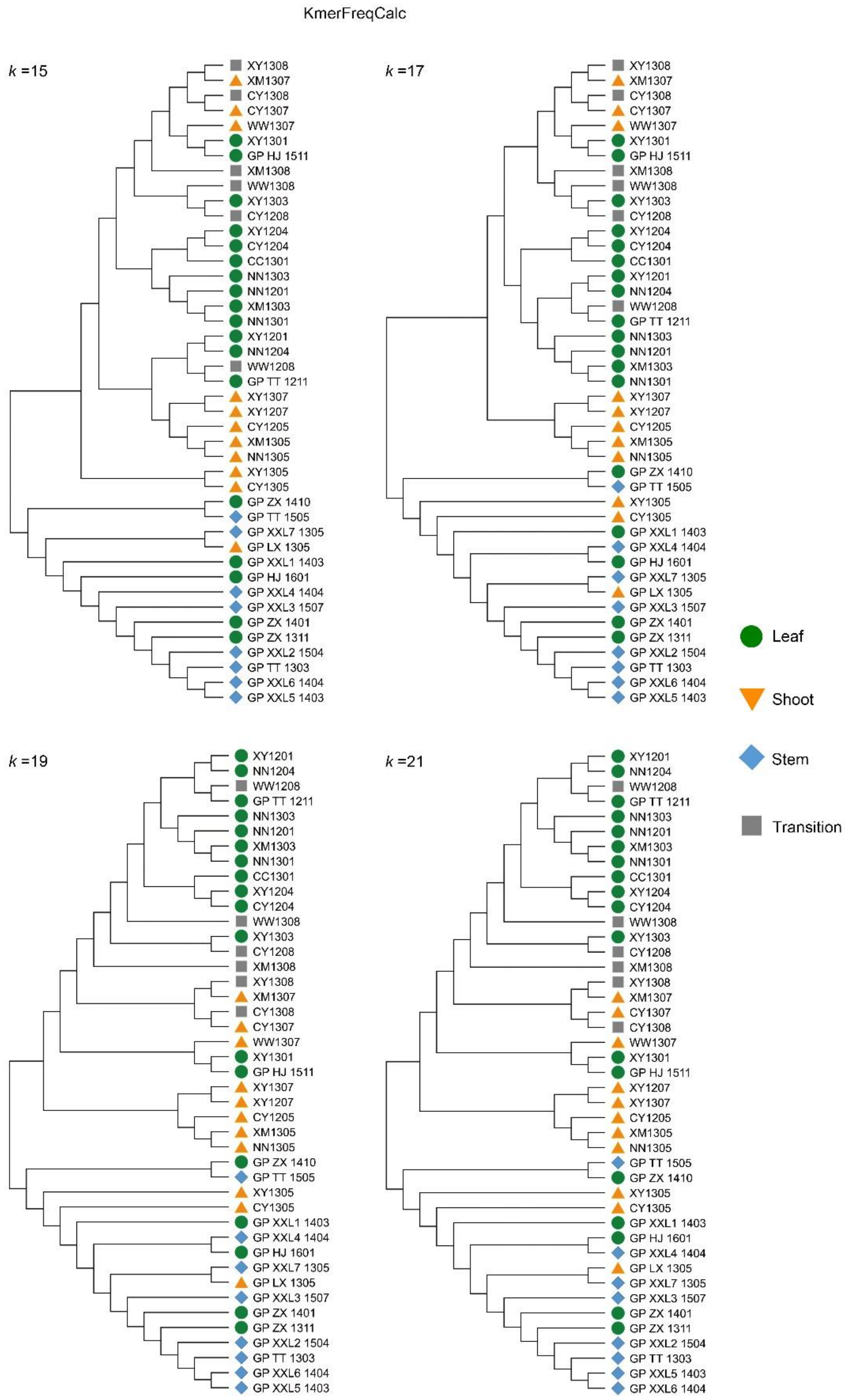

Figure S9 NJ-analysis of samples in QIN dataset based on Jaccard distance calculated by KmerFreqCalc with *k* = 15, 17, 19 and 21.
